# Differential gait adaptation patterns in Parkinson’s disease – a split belt treadmill pilot study

**DOI:** 10.1186/s12883-023-03321-4

**Published:** 2023-07-26

**Authors:** Meir Plotnik, Evyatar Arad, Adam Grinberg, Moran Salomon, Yotam Bahat, Sharon Hassin-Baer, Gabi Zeilig

**Affiliations:** 1 Center of Advanced Technologies in Rehabilitation, Sheba Medical Center, Tel Hashomer, Israel; 2 Department of Physiology and Pharmacology, Faculty of Medicine, Tel-Aviv University, Tel Aviv, Israel; 3 Sagol School of Neuroscience, Tel-Aviv University, Tel Aviv, Israel; 4 Department of Community Medicine and Rehabilitation, Umeå University, Umeå, Sweden; 5 Movement Disorders Institute and Department of Neurology, Sheba Medical Center, Tel Hashomer, Israel; 6 Department of Neurology and Neurosurgery, Faculty of Medicine, Tel-Aviv University, Tel Aviv, Israel; 7 Department of Neurological Rehabilitation, Sheba Medical Center, Tel Hashomer, Israel; 8 Department of Physical and Rehabilitation Medicine, Faculty of Medicine, Tel-Aviv University, Tel Aviv, Israel; 9 School of Health Professions, Ono Academic College, Kiryat Ono, Israel

**Keywords:** Parkinson’s disease, Asymmetry, SBTM, Arm swing, Kinematics, Gait pattern

## Abstract

**Background:**

Interventions using split belt treadmills (SBTM) aim to improve gait symmetry (GA) in Parkinson's disease (PD). Comparative effects in conjugated SBTM conditions were not studied systematically despite potentially affecting intervention outcomes. We compared gait adaptation effects instigated by SBTM walking with respect to the type (increased\decreased speed) and the side (more/less affected) of the manipulated belt in PD.

**Methods:**

Eight individuals with PD performed four trials of SBTM walking, each consisted of baseline tied belt configuration, followed by split belt setting – either WS or BS belt's speed increased or decreased by 50% from baseline, and final tied belt configuration. Based on the disease's motor symptoms, a 'worst' side (WS) and a 'best' side (BS) were defined for each participant.

**Results:**

SB initial change in GA was significant regardless of condition (*p* ≤ 0.02). This change was however more pronounced for BS-decrease compared with its matching condition WS-increase (*p* = 0.016). Similarly, the same was observed for WS-decrease compared to BS-increase (*p* = 0.013). Upon returning to tied belt condition, both BS-decrease and WS-increased resulted in a significant change in GA (*p* = 0.04). Upper limb asymmetry followed a similar trend of GA reversal, although non-significant.

**Conclusions:**

Stronger effects on GA were obtained by decreasing the BS belt’s speed of the best side, rather than increasing the speed of the worst side. Albeit a small sample size, which limits the generalisability of these results, we propose that future clinical studies would benefit from considering such methodological planning of SBTM intervention, for maximising of intervention outcomes. Larger samples may reveal arm swinging asymmetries alterations to match SBTM adaptation patterns. Finally, further research is warranted to study post-adaption effects in order to define optimal adaptation schemes to maximise the therapeutic effect of SBTM based interventions.

**Supplementary Information:**

The online version contains supplementary material available at 10.1186/s12883-023-03321-4.

## Background

Parkinson's disease (PD) is a neurodegenerative disorder. As the disease progresses it disrupts the patient's motor skills and causes a notable gait asymmetry, most likely stemming from asymmetrical neural dopaminergic degeneration of the nigrostriatal pathways [1]. Asymmetrical PD gait is expressed, for example, by both step length [2] and swing time [3] asymmetries, which were also implicated with the prevalence of the freezing of gait symptom and falls among persons with PD [4, 5]. Thus, in recent years, asymmetry has been a target for intervention in PD and other neurological disorders patients, utilising neural plasticity by means of conventional physiotherapy [6] or by the use of split belt treadmills (SBTM) [4, 7–9].

A SBTM is a useful tool to study neural adaptation mechanisms addressing bilateral function of gait, i.e., gait asymmetry (GA) and left–right stepping coordination [10, 11]. By inducing uneven speeds to the two belts, participants are forced to adapt their gait to the changing conditions by altering stance/swing times relations and thus, step length [12–14]. These changes are different for each leg, thus result in a consequent GA modification [10, 11, 14]. Often, in studies, participants are exposed to the split belt walking condition for a duration of 5–15 min, known as the 'adaptation period', which allows them to gradually adapt to the new walking pattern. Following the adaptation period, the belt speeds are reset back to equal speeds and effects of post-adaptation become apparent (i.e., induced change in GA) [4, 14–23]. In the case of intensive SBTM training programs, after-effects may be evident for weeks or months [24, 25]. SBTM walking can be administrated in different ways, i.e., increasing the right belt speed, decreasing it, or manipulating the left belt’s speed. In cases where disease’s motor symptomology is asymmetric, complexity increases when changes are made with reference to the more\less affected side. A recent systematic review [9] aimed to summarise the existing evidence on SBTM paradigms and effects on gait in PD compared to healthy controls. It concluded with a strong recommendation for a standardisation of current SBTM protocols, due to a large variation across studies in methodological aspects such as SB intervention parameters. The aim of this pilot study is to systematically compare the adaptation patterns associated with split-belt walking, with respect to the type of manipulation (increase\decrease speed) and side of the manipulated belt in reference to PD-related asymmetry. We hypothesised that stronger adaptation effects would be observed by augmenting the initial asymmetry by either reducing the belt's speed of the less affected side or increasing the speed of the more-affected side.

## Methods

### Participants

Individuals with PD were recruited after being referred by the Movement Disorders Institute at Sheba Medical Center, Ramat-Gan, Israel. Inclusion criteria were age: 40–80 years; PD diagnosis no more than ten years prior to testing; capable of walking without walking aids, stages 1–3 on the Hoehn and Yahr scale [26]; Montreal cognitive assessment (MOCA) score ≥ 22 [27] and a maximal score of 2 in the unified Parkinson’s disease rating scale (UPDRS) [28], q.29 and q.30 (indicating that the participant walks independently, fairly easy and doesn’t experience spontaneous loss of balance).

Participants were excluded if they had undergone brain surgery; if they exhibited motor fluctuations or dyskinesia; previous surgical procedures involving the lower limbs or other orthopedic or neurological problems affecting gait. Eight PD participants met the criteria and participated in the study (See Table 1 for demographic, clinical and gait speed data). The study was approved by the Ethical Committee of Human Studies at the Sheba Medical Center (reference number: SMC-9407–12). All participants provided written informed consent according to the Declaration of Helsinki prior to entering the study.

**Table 1:** Demographic, clinical and gait speed data

Participant	Sex	Age (y)	Weight (Kg)	Height (m)	MOCA score	UPDRS (Part III)	OG gait speed (m/s)	SBTM reference speed (m/s)
**P1**	M	51	68	1.72	22	17	1.36	0.95
**P2**	M	63	56	1.70	25	26	1.38	1
**P3**	M	68	103	1.98	27	35	1.36	1
**P4**	M	71	63	1.68	24	19	1.48	0.9
**P5**	M	67	92	1.78	9	22	1.2	1
**P6**	M	71	70	1.80	20	25	1.51	1
**P7**	M	73	92	1.80	28	34	1.42	1
**P8**	M	47	61	1.92	30	13	1.37	1
**Mean (SD)**	-	63.9 (9.1)	75.6 (16.3)	1.80 (0.1)	23.1 (6.1)	23.9 (7.3)	1.39 (0.09)	0.99 (0.03)

*Abbreviations:* *MOCA* Montreal cognitive assessment, *UPDRS* Unified Parkinson’s Disease Rating Scale, *OG* Over ground, *SBTM* Split-belt treadmill

### Apparatus

A split-belt instrumented treadmill (SBTM) (R-Mill, ForceLink, The Netherlands) equipped with force plate sensors, installed in a virtual reality based gait laboratory (V-Gait, Motek Medical, the Netherlands) was used. In the preset study no visual scenery was displayed. A motion capture system (Vicon, Oxford, UK) captured kinematic data from an array of passive markers attached to participants’ body while walking. Sampling rate of markers and force plate data was 120 Hz. A harness was worn by the participants during walking to prevent falls. It did not interfere with walking nor supported the participants’ body weight.

### Procedure

All participants were assessed in a single session while “ON” their anti-PD medications. To achieve this, we attempted to start the gait trials about 1–1.5 h after medication intake and confirming with the participant that they are in 'full ON'.

In this work we attempted to study the effects of SBTM walking, while participants were walking in their natural comfortable pace. In order to define this reference speed, we held preparatory walking trials that included the following stages:

- Over Ground Walking – Each participant performed four trials of the 10 m walking test (10MWT) in continuum [29]. Concisely, participants were asked to walk back and forth (without stopping) between the edges of a 24 m long corridor in their own comfortable self-selected pace until they were asked to stop. The experimenter used a stopwatch to time the duration by which a distance of 10 m was covered. Based on these measurements, the over ground gait speed was estimated.
- The participants were then acclimatised to walk on a treadmill with the aim to define the reference comfortable walking speed. The over ground walking speed was used as a basis for adjustments. During the adjustment, if a participant indicated the speed was not to his/her convenience, 0.1 m/s increments were used to fine tune the TM's speed. Reference speed limits were also defined so it could not exceed 1 m/s. The mean value (± SD) of self-preferred over ground walking speed was 1.38 ± 0.10 m/s. All but one participant (with over ground walking speed of 1.37 m/s) requested to reduce the treadmill speed, two of them to speed values lower than 1 m/s (i.e., 0.9, 0.95 m/s) and other to values greater or equal to 1 m/s. According to our reference speed limits the latter were introduced with reference speed of 1 m/s.
- Following the determination of the reference speed, participants performed four trials of SBTM walking presented in random order, separated by five minutes of seated rest. These trials were consisted of two minutes of walking with the two belts moving at the same speed (i.e., the reference speed – ‘tied belt’ (TB) configuration), this period was termed 'baseline period' (BL) and was followed by five minutes during which the two belts ran at different speeds ('split-belt' configuration). Finally, the belts moved in TB configuration for additional three minutes. The four types of trials differed from each other in terms of modified TM belt side, and in whether the belt's speed was increased or decreased by 50% relative to the reference speed.

In addition, we calculated the sum of scores of UPDRS-III (items 20 to 26 – items which refer to resting tremor, action or postural tremor, rigidity, finger taps, hand movements, rapid alternating movements of the hands and leg agility, respectively) for the right and the left side of the body separately. The higher and lower scoring sides were defined as the 'worst' and 'best' sides, respectively. An offline analysis was then performed in order to post-categorise the trials according to participants’ UPDRS asymmetry, thus defining four SB conditions: Best side decrease (BSD), worst side increase (WSI), worst side decrease (WSD) and best side increase (BSI). A post hoc analysis confirmed that carry-over after-effects between consecutive trials were negligible. We also defined matching conditions as pairs of two SB configurations with similar bilateral fast-slow relations, i.e. BSD – WSI and WSD – BSI. This classification allowed discriminating between SB configurations that resulted in initial GA exacerbation, i.e., BSD/WSI, to the other matching conditions which achieved the opposite effect.

### Data analysis and outcome measures

All calculations and analyses were done using custom MATLAB graphical user interface and scripts. The output measurements of this study are divided into three tiers. In the first tier are the basic measurements, later used to derive more advanced, higher tier, parameters. Detection of gait events (i.e., heel-strike and toe-off) was performed using the TM force plates (FP) [5]. FP data were low pass filtered using a 4^th^ order Butterworth filter with a dynamic cutoff frequency using the residual method [4]. Shoulder movement in the sagittal plane was calculated as the angle between the arm and a vertical reference line from the shoulder to the ground [6]. Tier 2 of the parameters included step length and arm swing amplitude. Step length was defined as the anterior–posterior distance between the two heel markers consequent to the heel strike (HS) of the measured leg [7]. Arm swing amplitude were calculated as the difference between shoulder angles at maximal anterior flexion and posterior extension of each arm. Tier 3, the last layer of calculated parameters utilised all of the above: Step length asymmetry was calculated as:

1 $$Step\;length\;asymmetry=\frac{Left\;step\;length-Right\;step\;length}{Left\;step\;length+Right\;step\;length}$$

As PD is frequently more pronounced in one hemisphere [10], the clinically affected side was taken into account in the analysis. Therefore, outcome measures were normalised in reference to the best\worst side as determined by the UPDRS score.

We define gait asymmetry (GA) with reference to the more (‘worse’) and less (‘best) affected sides in terms of PD signs. Therefore, Eq. 1 was modified as follows:

2 $$GA=\frac{Best\;side\;step\;length-Worst\;side\;step\;length}{Best\;side\;step\;length+Worst\;side\;step\;length}$$

Upper limb asymmetry (ULA) was calculated as:

3 $$ULA=\frac{Best\;side\;arm\;swing\;amplitude-Worst\;side\;arm\;swing\;amplitude }{Best\;side\;arm\;swing\;amplitude +Worst\;side\;arm\;swing\;amplitude}$$

Where arm swing amplitude is defined by the range of motion (in degrees) calculated as difference between the arm being in the most anterior position and the arm being at the most posterior position during the gait cycle. Recorded kinematic data were segmented into five periods: 1) Baseline (BL), 2) SB early adaptation (first 30 s of SB; EA), 3) SB late adaptation (last 30 s of SB; LA), 4) TB early post-adaptation (first 30 s after returning to TB; EPA), 5) TB late post-adaptation (last 30 s; LPA). Each period was analyzed separately for the different parameters’ means, standard deviations and coefficients of variance.

### Statistical analysis

Due to the small sample size, non-parametric statistics were applied. All four baseline measurements (one for each SB configuration) were compared by multiple Wilcoxon signed rank tests, to ensure a similar starting point for all four conditions in terms of GA. Step length and arm swing asymmetries were compared from BL to LPA (five stages) by means of a Friedman test for repeated measures, for each SBTM condition separately. Post hoc analysis was performed using Wilcoxon signed rank tests, corrected for multiple testing by the Bonferroni correction. Comparisons of interest were: BL to EA, EA to LA, EPA to BL, LPA to EPA, LPA to BL. Between condition comparisons were also performed, specifically comparing EA and EPA between all SBTM conditions with independent Mann–Whitney U tests. Significant level was set at α < 0.05. Statistical analysis was performed using IBM SPSS 20.0.

## Results

### UPDRS laterality and gait asymmetry

Baseline GA values were calculated for each participant as the mean of all four median BL segments. UPDRS Asymmetry mean value (± SD) was -0.104 ± 0.424 (range: (-1) – 0.33). Mean baseline GA values were 0.005 ± 0.01 (range: (-0.005 – 0.02). Shorter step length values were observed on the side also defined as the more affected side (i.e., in terms of PD motor symptoms) in five out of the eight participants. A post-hoc comparison between all of the conditions’ baselines showed no difference in GA (*p* = 0.774).

### Step length asymmetry – the effect of the different SBTM conditions

**Fig. 1:**
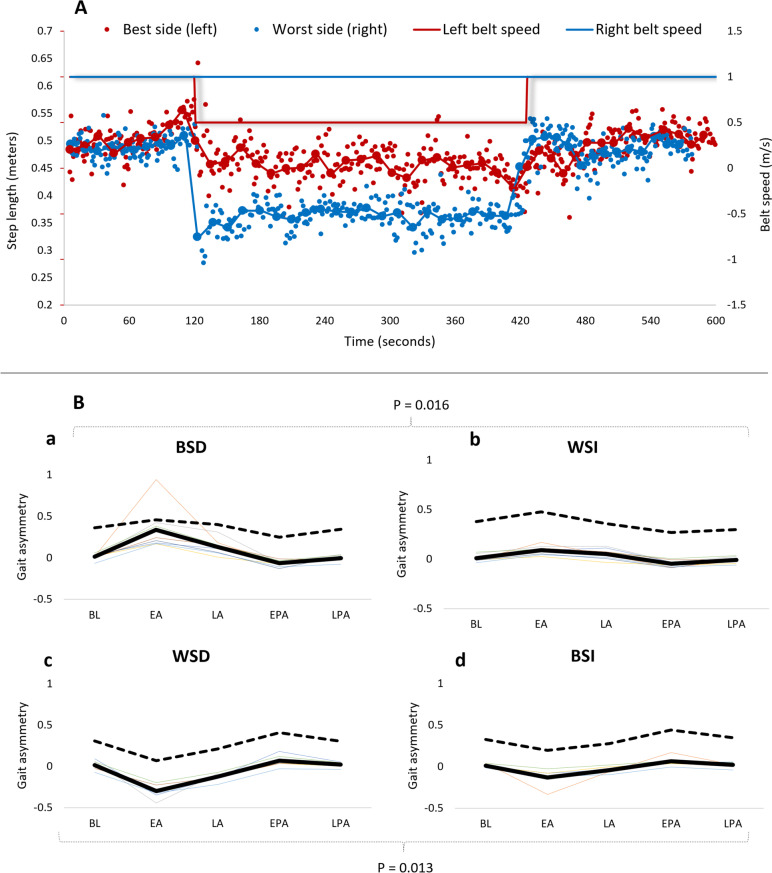
**A** Step length values during a split belt treadmill trial. Step length values (red- left; blue-right; left ordinate) from a complete 10 min trial are depicted. This participant had more sever Parkinsonian signs on the right side of the body (‘worst side’). After two minutes of baseline walking (BL) with tied belts (TB), the speed of left belt was reduced by 50% (‘best side decrease’ – BSD; right ordinate). Gait asymmetry (GA) was exacerbated for the next 5 min of split belt walking more in the early adaptation phase (EA, e.g., 120–150 s) compared to the late adaptation (LA, e.g., 390–420 s’ – see also Fig. 2B). The trial ended with 3 more minutes of tied belt walking. It can be seen that GA was reversed in the early post adaptation stage (EPA, e.g., 420 – 450 s’), but the effect did not last and subsequently returned to resemble BL GA in late post adaptation (LPA). Big dots represent the averages of step lengths in each side, connected by a trend-line. None of these points contain mixed data from different belt conditions. Few right step length values are missing from the end of the trial due to technical problem. **B **Upper and lower limbs asymmetry at five epochs for four split-belt conditions (see methods for details). Upper panels correspond to early increase in gait asymmetry (GA) of baseline asymmetry by either decreasing the belt speed on the less-affected side (a; Best side decrease) or by increasing the belt speed on the more affected side (b; Worst side increase). The lower panels correspond to early increase in GA of baseline asymmetry towards the opposite direction by either decreasing the belt speed on the more affected side (c; Worst side decrease) or by increasing the belt speed on the less-affected side (d; Best side increase). Adaptation asymmetry in the upper limbs displayed a similar trend compared to lower limb GA, although non-significant for all comparisons (Wilcoxon signed rank test, *p* ≥ 0.051). Traces represent step length asymmetry for each participant (thin traces) as well as mean values (bold trace) and mean upper limbs asymmetry (dashed traces). BL – baseline, EA – early adaptation, LA – late adaptation, EPA – early post-adaptation, LPA – late post-adaptation

A typical example of step length data from a BSD trial from one participant is depicted in Fig. 1A. It can be seen that the initial GA is further aggravated during the SB mode, and that in the post adaptation period, GA is reversed, though the effect subsides shortly after. This was true for all conditions for all participants: GA measured in the last 30 s in TB setting (i.e. LPA stage) was not statistically significant for any condition compared to BL (*p* ≥ 0.200).

Summary of GA values across the various stages and conditions are presented in Fig. 1B and in Table S1 (Supplementary material).

**Fig. 2:**
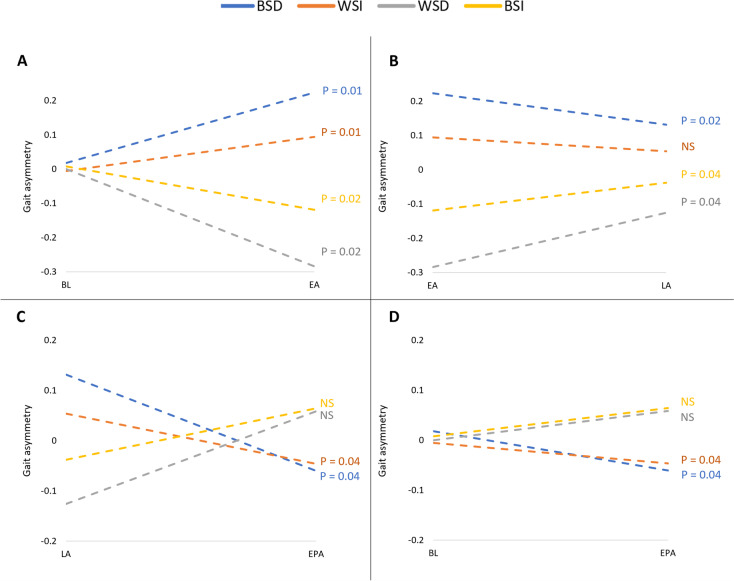
Median Split-belt (SB) gait asymmetry (GA) throughout four split-belt conditions (BSD, WSI, WSD and BSI, depicted colours, see key). **A** *Initial effect.* GA changes between baseline (BL) and early adaptation (EA) were significantly affected regardless of SB condition. The mean values (± SEM) of the percentile change in GA, from BL, were 34.0 ± 21.2% and 11.3 ± 8.5%, when decreasing and increasing belt speed, respectively (nonparametric testing: *p* = 0.0078; lumping both 'decrease' trials for comparison with both 'increase' trials). **B** *Adaptation.* GA changes between early adaption (EA) and late adaptation (LA) in three out of four conditions (all except WSI) were found significant. **C** *Post-adaptation.* GA changes between late adaptation (LA) and early post-adaptation (EPA) showed a reversal effect. **D** *Therapeutic effect.* GA in baseline (BL) and early post-adaptation (EPA). BSD/WSI matching conditions showed a significant change in GA from BL to EPA. BSD – Best side decrease, WSI – worst side increase, WSD – Worst side decrease, BSI – best side increase, NS – non-significant

There were also greater GA adaptation effects observed in conditions which exacerbated the initial asymmetry (Fig. 2). Specifically, BSD had a more pronounced GA at EA than WSI (*p* = 0.016) and WSD had a more pronounced GA at EA than BSI (*p* = 0.013).

SB initial effect, (i.e., EA compared to BL; Fig. 2A), was significant regardless of condition (*p* ≤ 0.02).

In terms of SB adaptation (i.e., LA compared to EA), the initial increase in GA was significantly attenuated in three out of four conditions (all except WSI; *p* ≤ 0.04; Fig. 2B). GA changes between LA and EPA showed a reversal effect (Fig. 2C). Finally, BSD/WSI matching conditions showed a significant change in GA from BL to EPA (*p* = 0.04; Fig. 2D).

### Upper limb asymmetry within conditions

ULA values, in general, were higher than GA. During BL, median ULA value was 0.37 (range: -0.16 – 0.67), which was laterally consistent with participants’ UPDRS asymmetry (i.e., affected side = less arm swinging, except for one case). Adaptation asymmetry in the upper limbs followed a similar trend compared to lower limb GA, although non-significant for all comparisons (*p* ≥ 0.051).

## Discussion

The present study, for the first time to our knowledge, evaluates gait adaptation patterns in all four SBTM conditions. We found that stronger adaptation effects are obtained by decreasing a belt’s speed, as opposed to increasing the speed of the opposite belt. This distinction is rarely addressed in SBTM studies.

Nenhoe-Mahabier et al. found no significant differences between BSI and WSI and treated the two SB conditions interchangeably [30]. Fasano et al. distinguished between two non-matching SB conditions, BSD and WSD, and found BSD the most effective condition for GA adaptation, in agreement with our results [4]. Further to their study, we observed that the matching condition (WSI) had weaker effects on GA.

We found that the BSD/WSI matching conditions bears the highest potential impact on the post adaptation period (compare panels A & D in Fig. 2), by first inducing and increase in baseline asymmetry during the early adaptation period, i.e., by placing the leg with the longest step on the relatively slower belt (BSD), thereby initially producing a longer step length in response to the asymmetric belt speed [31]. Even though our participants were relatively symmetric at baseline, when asymmetry was induced via SBTM intervention, a change was observed in the opposite direction (Figs. 1 and 2).

Differences *within* pairs of matching conditions were further observed (e.g., BSD compared to WSI), both as a better SB initial effect (Fig. 2A) and during adaptation stage (i.e., comparing LA with EA) (Fig. 2B). As individuals are walking in their comfortable pace, reducing a belt’s speed is likely to keep them in their “comfort zone”, thus leaving room for appropriate gait modification. Increasing a belt’s speed, however, introduces a less manageable situation, in which one is asked to react to a perturbation that may exceed his/her physical limitations. Another explanation stems from left–right speed ratios. Increasing one belt’s speed by 50% results in a 2:3 speed ratio, while decreasing a belt’s speed by 50% results in a 1:2 ratio, a more aggressive intervention which may result in a more robust change to the gait pattern.

Interestingly, we found that SBTM walking produced similar trends of adaptation patterns in arm swinging asymmetry (Fig. 1B), presumably reflecting spinal inter-segmental neuronal synchronisation between upper and lower limb central pattern generators (CPG) [29]. Specifically, the BSD/WSI matching condition resulted in a more symmetrical arm swinging in the first 30 s after returning to TB setting (i.e., EPA), compared to BL. Input to the CPG is delivered partly by supra-spinal structures, such as the cerebellum and basal ganglia. It is likely that the split-belt intervention induces a neural change at the level of these structures [18, 21]. Moreover, the sub thalamic nucleus and dopaminergic motor-related systems exert less influence on the executive circuitry responsible for arm movements, but have a preferential action on those for the gait cycle of the lower limbs [23]. Such notion may serve as an explanation for the relatively higher level of dysfunction of the upper limbs in terms of asymmetry, relatively to the lower limbs in our cohort. On the other hand, gait adaptation is a form of supervised, error-based motor learning, attributed mainly for the cerebellum [24], which may explain the similar upper/lower limb trends.

None of the outcomes was significantly different in the LPA period, compared to BL. Moreover, the trends for both step length and arm swing asymmetries were consistently directed back towards BL values. This absence of post-adaptation long-term effects might be attributed to the relatively short SB duration and would warrant future studies aiming to establish a more beneficial and long-lasting therapeutic effect.

Two major limitations must be acknowledged. The first is the small sample size. This was an exploratory study and results should therefore be interpreted with caution. Given that persons with PD show high amounts of variability in their gait features, a larger scale investigation is warranted in order to make the results generalisable. Secondly, our sample was comprised of individuals with relatively symmetrical step-lengths. While this is not an optimal baseline to assess SB-induced asymmetry change, the similar trends observed in the upper limb suggest that similar effects would have been observed for initially-asymmetrical individuals. This, however, should be corroborated in future studies and potentially by including a control group(s). In the current study design, each participant provided their own baseline, resulting in paired comparisons of SBTM manipulation effects presumably providing a sufficient comparator. Still, future studies, that will include a control group, might be required to confirm our finding on the superiority of BSD among other asymmetrical patient groups. For example, involving a group of persons with lower limb amputation, whose observed asymmetry is of orthopedic origin rather than neurodegeneration. Our results, although exploratory, provide insights regarding strategies for the implementation of SBTM interventions when attempting to address gait asymmetry in persons with neurological deficits, for either research or physiotherapy [4, 7, 11, 13, 15, 16, 24]. Particularly, we posit that the speed of the belt of the less affected side should be decreased in order to obtain a stronger adaptation, and consequently a stronger post-treatment effect. The latter, however, could not be confirmed due to short adaptation and post adaptation periods used. Our results should also be corroborated on initially asymmetrical individuals, which unfortunately was not the case with our cohort.

## Conclusions

Our results demonstrate that in order to achieve stronger SB adaptation effects on GA, the speed of the best side belt should be decreased, rather than increasing the speed of the worst side. Future clinical studies would benefit from considering such methodological planning of split-belt sessions for maximising intervention outcomes. In addition, studies with larger sample sizes are needed in order to further elucidate the residual influence of SB interventions on arm swinging. Finally, post-adaption effects, while not observed in the current investigation, must be further explored by means of longer-duration interventions, in order to maximise the therapeutic outcomes of SBTM-based treatments.

## Supplementary Information

**Additional file 1: Table S1.** Step length asymmetry values throughout the four trials (median [range]).

## Data Availability

The datasets generated during and/or analysed during the current study are available on reasonable request.

## Abbreviations

BSD: Best side decrease
BSI: Best side increase
CPG: Central pattern generators
EA: Early adaptation
EPA: Early post-adaptation
FP: Force plate
GA: Gait asymmetry
HS: Heel strike
LA: Late adaptation
LPA: Late post-adaptation
MOCA: Montreal cognitive assessment
OG: Over-ground
PD: Parkinson’s disease
SB: Split-belt
SBTM: Split-belt treadmill
TB: Tied-belt
TM: Treadmill
ULA: Upper limb asymmetry
UPDRS: Unified Parkinson’s disease rating scale
WSD: Worst side decrease
WSI: Worst side increase
