# Supplementary material for: Differential gait adaptation patterns in Parkinson’s disease – a split belt treadmill pilot study
Source: BMC Neurol. 2023 Jul 26;23:279. doi: 10.1186/s12883-023-03321-4 (PMC10369736; doi:10.1186/s12883-023-03321-4)
Supplement: Supplementary file 1 — Additional file 1: Table S1. Step length asymmetry values throughout the four trials (median [range]). [file 12883_2023_3321_MOESM1_ESM.docx]

**Supplementary material**

*Table S-1.* Step length asymmetry values throughout the four trials (median [range])

|  | BL | EA | LA | EPA | LPA |
| --- | --- | --- | --- | --- | --- |
| BSD | 0.02  [(-0.07) – 0.06] | 0.22 ^a^  [0.17 – 0.94] | 0.13 ^b^  [0.01 – 0.31] | -0.06 ^c^  [(-0.13) – (-0.01)] | 0  [(-0.08) – 0.04] |
| WSI | -0.01  [(-0.04) – 0.07] | 0.09 ^a^  [0.03 – 0.17] | 0.05 ^b^  [(-0.03) – 0.13] | -0.05 ^c^  [(-0.09) – 0] | 0  [(-0.06) – 0.03] |
| WSD | 0  [(-0.07) – 0.09] | -0.28 ^a^  [(-0.44) – (-0.19)] | -0.13 ^b^  [(-0.22) – (-0.07)] | 0.06  [(-0.03) – 0.18] | 0.03  [(-0.04) – 0.06] |
| BSI | 0.01  [(-0.02) – 0.04] | -0.12 ^a^  [(-0.34) – (-0.02)] | -0.04 ^b^  [(-0.1) – 0.02] | 0.06  [0 – 0.17] | 0.02  [(-0.04) – 0.06] |

**^a^** BL to EA significant change.

**^b^** EA to LA significant change.

**^c^** BL to EPA significant change.

**Abbreviations:** BSD – best side decrease; WSI – worst side increase; WSD – worst side decrease; BSI – best side increase; BL – baseline; EA – early adaptation; LA – late adaptation; EPA – early post-adaptation; LPA – late post-adaptation.
